# Validation of SRS MapCHECK for CyberKnife patient-specific quality assurance: challenges with small cone sizes

**DOI:** 10.3389/fonc.2025.1667108

**Published:** 2026-01-08

**Authors:** Dohyeon Yoo, Taeho Kim, Hojae Kim, Sangmin Lee, Dong Wook Kim, Jin Sung Kim, Ho Lee, Hojin Kim

**Affiliations:** Department of Radiation Oncology, Yonsei Cancer Center, Heavy Ion Therapy Research Institute, Yonsei University College of Medicine, Seoul, Republic of Korea

**Keywords:** CyberKnife, stereotactic radiosurgery, SRS MapCHECK, patient specific quality assurance, angular correction, small field dosimetry

## Abstract

**Purpose:**

The CyberKnife system, designed for stereotactic radiosurgery (SRS) and stereotactic body radiotherapy (SBRT), considers SRS MapCHECK an effective tool for patient-specific QA (PSQA). This work evaluates the performance of SRS MapCHECK in CyberKnife PSQA, identifies potential sources of inaccuracy in PSQA analysis, and recommends accurate and reliable use of this tool for small fields.

**Methods:**

SRS MapCHECK is a promising tool for CyberKnife PSQA due to its compact size and high-resolution 2D detector. It enhances dosimetry accuracy by incorporating correction factors for field size and gantry angle. To validate its effectiveness, PSQAs were performed on CyberKnife plans with various fixed cone sizes for brain SRS cases. Static field dose delivery was tested by rotating robotic arm angles for different fixed cone sizes under various correction settings to assess the impact of these correction factors on dosimetric accuracy.

**Results:**

For PSQAs on 12 patient plans, statistically significant negative correlation (spearman correlation *ρ* = -0.87, *p-value* < 0.0001) was found between cone size and the absolute dosimetric error. Group analysis confirmed significantly larger under-measurements for small fixed cones (≤12.5 mm; mean difference: -5.66 ± 4.31) compared to large cones (≥15 mm; mean difference: -0.16 ± 0.80; unpaired t-test, *p-value* = 0.0062). Static dose delivery experiments revealed that these discrepancies were primarily linked to the correction factors used in SRS MapCHECK, which showed limited or negative effects for cones ≤12.5 mm despite stabilizing measurements for larger fixed cones (≥15 mm).

**Conclusion:**

This study evaluates the suitability of SRS MapCHECK for PSQA of the CyberKnife system and highlights its limitations. It demonstrates that while SRS MapCHECK is effective for larger fixed cone sizes, it does not ensure dosimetric accuracy for plans involving very small fixed cones.

## 1 Introduction

Stereotactic radiosurgery (SRS) and stereotactic body radiotherapy (SBRT) are characterized by the delivery of a high dose of radiation in one or a few fractions to small primary target or metastatic target volumes, which are often very small (1–6). These techniques require specialized strategies to ensure accurate dose delivery to the target volume, while sparing the surrounding healthy tissue. A non-coplanar treatment scheme can facilitate achieving the goal, whereas most conventional linear accelerators perform radiotherapy using coplanar schemes. CyberKnife (Accuray Incorporated, Sunnyvale, CA, USA) is specifically designed for SRS/SBRT, offering a non-coplanar treatment scheme by its robotic arm, combined with its unique patient motion tracking system (7, 8). To enhance treatment plan quality, it also employs a non-isocentric delivery scheme, which allows radiation beams to deviate from the isocenter. Additionally, CyberKnife provides various collimation options by varying the fixed cones from 5 mm to 60 mm and applying multi-leaf collimators (MLCs) with field sizes from 7.6 mm × 7.5 mm to 115.0 mm × 100.0 mm. For very small target volumes, small fixed cones are often preferred over MLCs to ensure treatment precision.

The non-coplanar and non-isocentric capabilities of the CyberKnife are highly beneficial for SRS/SBRT (9). However, precise dose verification is crucial as potential dosimetric and geometric errors can lead to severe consequences, such as missing the target volume and causing unnecessary exposure to healthy tissue (10–12). Therefore, rigorous dosimetric patient-specific quality assurance (PSQA) is essential to ensure accuracy and safety before actual treatment. Notably, the non-isocentric nature of CyberKnife cannot be adequately verified by quality assurance (QA) tasks any other than PSQA, further emphasizing the necessity of PSQA. In practice, however, limited solutions are available for the dosimetric quality assurance (DQA) of the CyberKnife system. A commercial system with two-dimensional (2D) array detectors composed of silicon diodes, such as Octavius (PTW, Freiburg, Germany), is suited for SRS/SBRT PSQA due to their high spatial resolution (typically less than 0.2 mm^3^), stable energy response to megavoltage photon beams, excellent dose linearity, and real-time readout capability (13). Nevertheless, these detectors have limitations, including dependencies on dose rate and dose-per-pulse, over-response to low-energy photons, and directional sensitivity. Moreover, their large size poses a collision risk with the CyberKnife’s robotic arm. Another common method in SRS/SBRT is a dose verification phantom (Standard Imaging Inc., Middleton, WI, USA), which combines ion chambers for absolute dosimetry and film for relative dosimetry (14, 15). Despite its accuracy, this method might be inefficient for the very small fields where PSQA cannot be assessed at once using film and ion chamber measurements simultaneously. To address these challenges, the SRS MapCHECK (Sun Nuclear Corp., Melbourne, FL, USA) has emerged as a promising solution for PSQA in SRS/SBRT (16–21). Infusino et al. (22) compared the SRS MapCHECK with GAFchromic films for various CyberKnife collimators and found it to be a reliable replacement for film dosimetry. Xu et al. (23) implemented and evaluated the SRS MapCHECK for robotic brain SRS/SBRT, concluding it was a viable option with high gamma passing rate. Other studies have focused on different high-resolution arrays, such as Ashraf et al. (24) using an angular correction methodology for CMOS array on the CyberKnife, highlighting the importance of angular dependency correction.

This device meets the high-precision requirements of SRS/SBRT and offers an efficient PSQA process by incorporating correction factors for various field sizes and beam angles. While many studies have reported the excellent performance of SRS MapCHECK in general, some have highlighted potential limitations in small-field applications, underscoring the importance of rigorous, system-specific validation. In particular, the reliability of the built-in correction factors provided by the SRS MapCHECK has never been thoroughly investigated for PSQA in CyberKnife treatments involving highly demanding small fixed cones. Therefore, this study conducted a focused evaluation of the SRS MapCHECK specifically for the CyberKnife system using small fixed cones with the following objectives:

- To investigate the dosimetry accuracy of the SRS MapCHECK in PSQA of the CyberKnife with fixed cones.
- To identify factors affecting the dosimetry accuracy of the SRS MapCHECK based on PSQA results.
- To propose practical recommendations for its reliable clinical implementations.

To enhance the robustness of the analysis, SRS MapCHECK measurements were performed for CyberKnife plans with various small fixed cone sizes, followed by statistical analyses to examine correlations between dosimetry accuracy and fixed cone size. In addition, a static field evaluation was conducted to assess the influence of the built-in correction factors of the SRS MapCHECK on dosimetry accuracy. By integrating these findings, clinically applicable recommendations were derived and presented for the accurate and reliable use of the SRS MapCHECK in CyberKnife PSQA.

## 2 Materials and methods

### 2.1 CyberKnife

**Figure 1:**
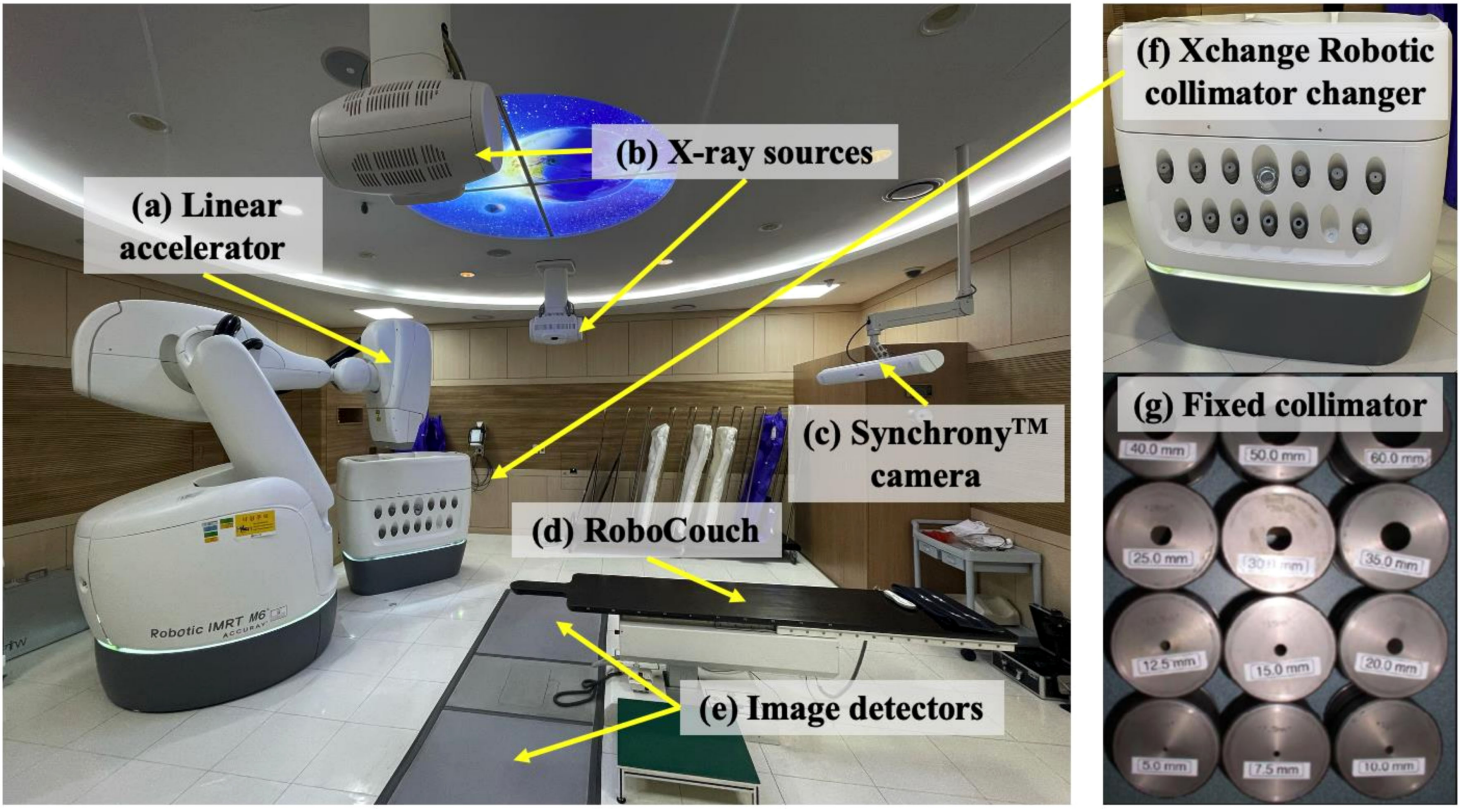
Overview of CyberKnife^®^ M6^™^ model from Accuray at Yonsei Cancer Center; **(a)** Linear accelerator mounted robotic arm, **(b)** X-ray source, **(c)** Synchrony™ camera, **(d)** RoboCouch, **(e)** Image detectors from x-ray source, **(f)** Xchanger Robotic collimator changer, and **(g)** different fixed collimator sizes from 5.0 mm to 60 mm.

**Figure 1** shows the CyberKnife^®^ M6™ system installed at Yonsei Cancer Center, Seoul, South Korea (25). A key component of this system is a linear accelerator mounted on a robotic arm capable of independent movement around six axes (**Figure 1a**). The patient positioning system, known as RoboCouch, adjusts the patient’s position using a robotic mechanism with five degrees of freedom (**Figure 1d**). The treatment table moves in three translational directions (Inferior/Superior, Left/Right, and Anterior/Posterior) and two rotational directions (Roll and Pitch). These capabilities enable irradiation from multiple angles, allowing a high dose to be delivered to the target volume while sparing surrounding healthy tissues (26).

The CyberKnife enables the Monte Carlo (MC) dose calculation algorithm for both fixed cones and MLC (27, 28). This algorithm is essential for enhancing treatment accuracy as it supports heterogeneity corrections to account for variations in tissue density, a capability that the alternative ray tracing algorithm for fixed cones and pencil beam algorithm for MLC lacks. Thus, in most cases, the MC dose calculation has been employed for both patient plans for treatment and PSQA plans for verification at YCC.

### 2.2 SRS MapCHECK specifications

**Figure 2:**
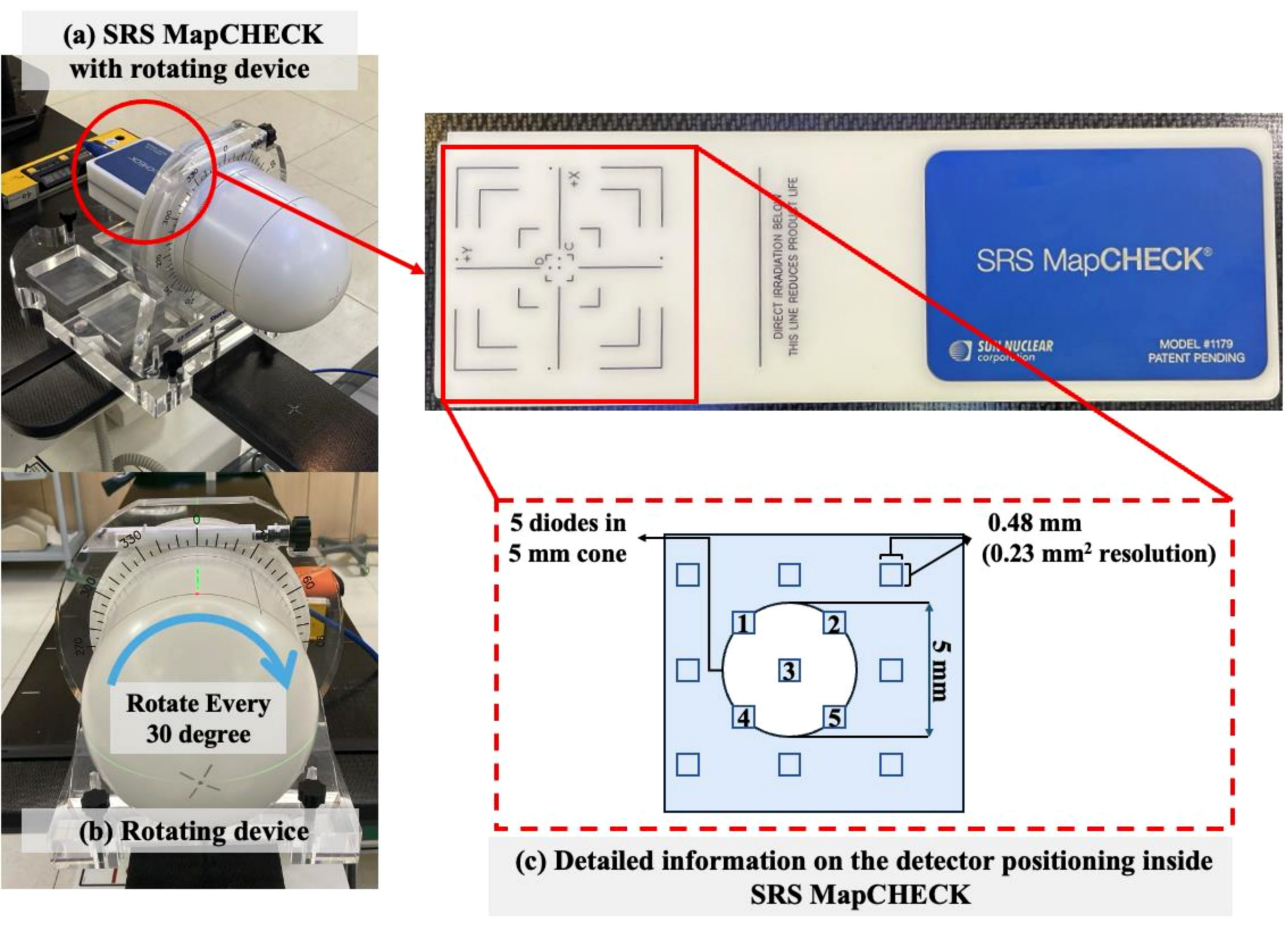
**(a)** The SRS MapCHECK device with rotating cover designed for small-field dosimetry and **(b)** detailed geometry of the rotating head, and **(c)** detailed information on the detector positioning inside SRS MapCHECK.

The SRS MapCHECK device for PSQA is detailed in **Figure 2a**. This two-dimensional detector array is designed specifically for small-field dosimetry in SRS/SBRT, with the device head rotating every 30° (**Figure 2b**). The device features a high-resolution silicon diode array, which is well-suited for measuring dose distributions in small fields with high precision. SRS MapCHECK comprises 1,013 detectors, each with a size of 0.48 mm × 0.48 mm and a resolution of 0.23 mm², providing high spatial resolution in the central region, with 5 diodes positioned in a 5 mm cone size (**Figure 2c**). A key feature of the SRS MapCHECK is its inclusion of correction factors for various field sizes and beam angles, which compensate for potential dependencies on field size and device orientation. This design makes it particularly suitable for use with high-precision radiation therapy devices like the CyberKnife. Additionally, its capability for real-time dose distribution verification enhances the efficiency of the PSQA process for SRS/SBRT. The correction methods for this device include small-field correction and gantry angle correction, utilizing two embedded sensors (Mother and Daughter sensors) to mitigate variations in dose response due to changes in tilt or orientation.

In this study, a two-step calibration process was required before using the SRS MapCHECK for the PSQA process. Both the Elekta Versa HD (Elekta AB, Stockholm, Sweden) and CyberKnife M6 were employed for the calibration process, in which the Versa HD was used for the initial calibration using a 6 MV energy. While minor spectral differences between manufacturers are inevitable, the overall beam quality is considered highly comparable, minimizing potential energy-dependent variations in the diode response. The first step involved relative calibration performed on the Versa HD, which was intended to normalize the sensitivity of the 1,013 individual diodes relative to each other, ensuring a uniform response profile across the array, rather than determining the absolute dose. Minor shifts in the overall array response due to spectral differences would not significantly affect this inter-diode normalization. The second step was the absolute calibration, performed on the CyberKnife M6 using the machine-specific reference field size (60 mm fixed cone). This step anchors the response of the calibrated array to the absolute dose delivered by the actual treatment beam quality. This methodology, particularly the use of a machine-specific reference field for the final absolute calibration, is consistent with the principles outlined in advanced dosimetry protocols such as AAPM Task Group 155 (TG-155), which specifically addresses the challenges of small-field dosimetry (29). A density correction factor of 1.2 g/cm^3^ was also applied to match the settings in the treatment planning system (TPS), ensuring accurate dose calculations under clinical conditions.

### 2.3 Experiment conditions

The study protocol was approved by the Ethics Committee/Institutional Review Board of Severance Hospital, Yonsei University College of Medicine, Seoul, Republic of Korea (IRB approval no. 4-2024-1314), which also determined that informed patient consent was not required for the retrospective analysis of patient images. This work conducted two types of experiments with fixed cones for SRS MapCHECK: PSQA and static delivery while changing gantry angles and correction factors. First, PSQA was performed on treatment plans for 12 brain tumor patients that utilized various cone sizes (5, 7.5, 10, 12.5, 15, 20, and 25 mm). Treatment plans were generated using the Accuray Precision treatment planning system (version 2.0.1.1, Accuray Incorporated, Sunnyvale, CA, USA) with the MC dose calculation algorithm at 1% uncertainty. Each PSQA plan was delivered to the SRS MapCHECK fixed on the couch, and the location of the detector was guided by 2D kV X-ray images with two metal fiducial markers embedded. Gamma passing rates and absolute dose differences between the TPS and measurements were compared using the SRS MapCHECK PSQA program with all correction factors on (“All check”) and the gamma index criterion of 3%/3 mm with a 10% low-dose threshold. The second experiment was performed to verify if the correction factors for various field sizes and angles provided by SRS MapCHECK function appropriately. SRS MapCHECK measurements were conducted by rotating the angles of the robotic arms from 0° to 330° in 30° increments for measurements, while changing the fixed cone sizes to 7.5, 10, 12.5, 15 and 60 mm at a source-to-axis distance (SAD) of 80 cm. The measured dose data were then analyzed using four different correction settings: “All check” (all the correction factors activated), “No check” (none of the correction factors activated), “Angular correction only” (only angular correction activated), and “Field size correction only” (only field size correction activated). Furthermore, to independently verify the accuracy of the TPS calculations, we performed our in-house PSQA tasks, as a comparative analysis, consisting of the absolute point dose measurement using Exradin A16 ionization chamber (Standard Imaging Inc., WI, USA) and the quantification of gamma passing rate using GAFchromic EBT3 film (Ashland Specialty Ingredients G.P., Bridgewater, NJ, USA). The measurement was conducted with a stereotactic dose verification phantom (Standard Imaging Inc., WI, USA). Film measurements were evaluated using a gamma index criterion of 3%/3 mm with a 10% low-dose threshold, alongside a point dose comparison.

## 3 Results

### 3.1 Measurement of PSQA plans

**Figure 3:**
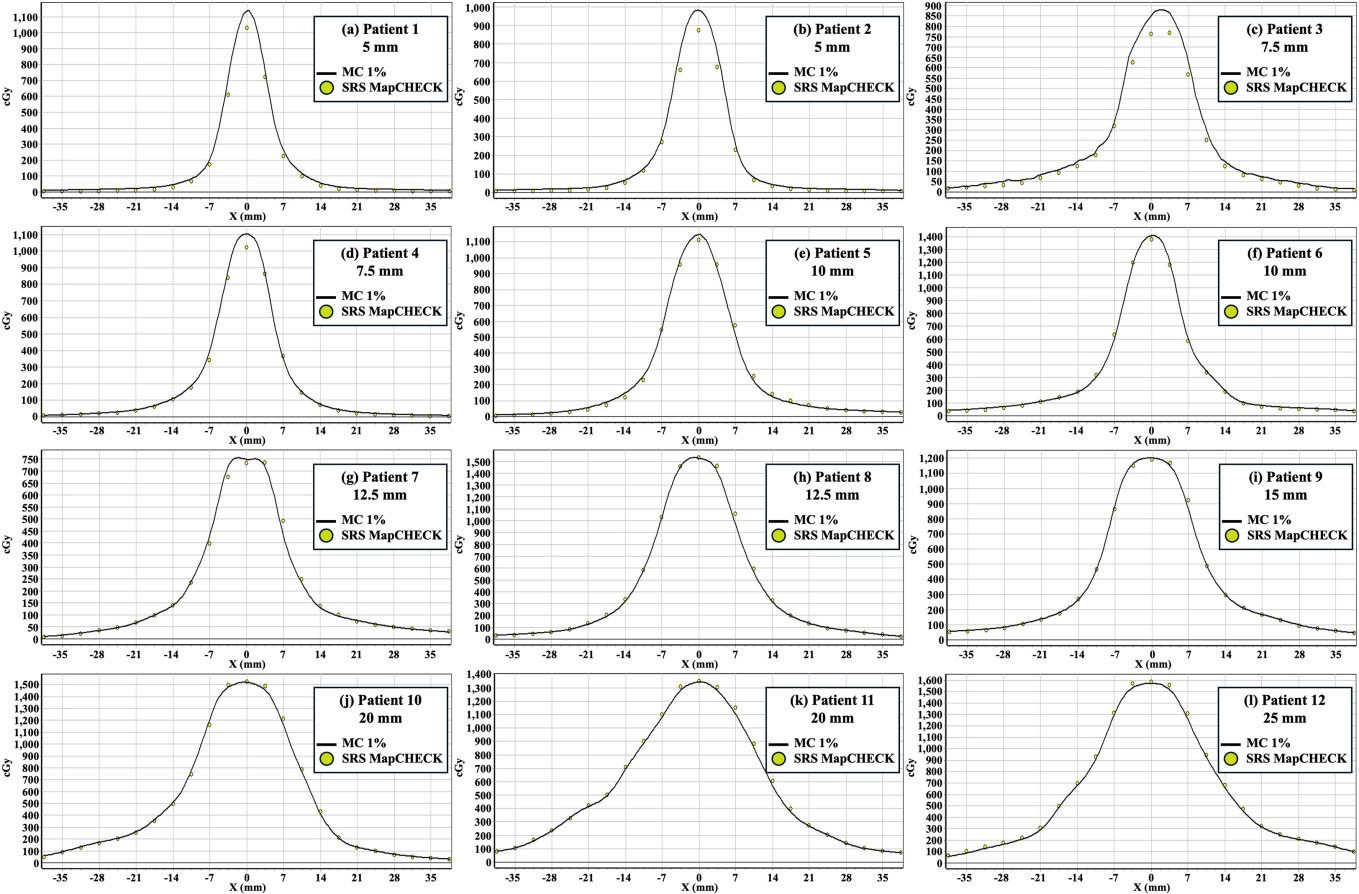
Comparison of dose profiles between SRS MapCHECK measurements (yellow dots) and Monte Carlo (MC) calculations from the treatment planning system (TPS) (solid line). The profiles are for 12 brain tumor patients treated with the CyberKnife using various cone sizes: **(a, b)** 5 mm, **(c, d)** 7.5 mm, **(e, f)** 10 mm, **(g, h)** 12.5 mm, **(i)** 15 mm, **(j, k)** 20 mm and **(l)** 25 mm.

**Figure 3** shows the dose profile of the SRS MapCHECK measurements, relative to the TPS calculations for 12 brain tumor patients treated with the CyberKnife system using different cone sizes (5, 7.5, 10, 12.5, 15, 20, and 25 mm). It revealed that the dosimetric differences increased as the fixed cone size decreased. The discrepancy was clearly pronounced for the plans using fixed cones smaller than 10 mm. **Table 1** lists the numerical results of the gamma passing rate and point dose from the SRS MapCHECK measurements. In addition, the phantom-based PSQA results were provided for comparison. Importantly, the phantom-based PSQA results showed an absolute point dose error less than 5% and a gamma passing rate greater than 90% (3%/3 mm), which was greater than 95% for most cases. This implies that the TPS calculations were reliable and unlikely to be a source of the dosimetric errors in PSQA measurements. For the SRS MapCHECK measurements, however, the dosimetric errors were explicit in the point dose errors. The discrepancies decreased significantly for large cones, such as 0.14% for a 12.5 mm cone and 0.37% for a 20 mm cone. In contrast, the point dose differences were -9.13% and -10.92% for the 5 mm fixed cones, and -10.49% and -7.35% for the 7.5 mm fixed cones, respectively. Compared to the point dose, the gamma passing rates remained consistently high, resulting in a smaller standard deviation than that from the phantom-based PSQA. The plans using extremely small cones must have very small target volumes. In such conditions, the large discrepancy in small target volumes could be masked in the gamma index, thus possibly overestimating the plan deliverability.

**Table 1:** Comparison of gamma index criteria (3%/3 mm) and central axis (CAX) dose between measurement and TPS for 12 brain tumor patients using different cone size (5, 7.5, 10, 12.5, 20, and 25 mm) using SRS MapCHECK and phantom based QA.

Cone size	SRS MapCHECK		*Phantom based QA	
	Gamma index (3%/3 mm)	% difference	Gamma index (3%/3 mm)	% difference
Patient 1 (5 mm)	100	-9.13%	90.76	0.90%
Patient 2 (5 mm)	100	-10.92%	99.63	2.37%
Patient 3 (7.5 mm)	98.2	-10.49%	99.99	-3.49%
Patient 4 (7.5 mm)	100	-7.35%	100	1.49%
Patient 5 (10 mm)	100	-3.09%	90.51	3.68%
Patient 6 (10 mm)	100	-2.25%	99.93	0.84%
Patient 7 (12.5 mm)	100	-2.16%	93.17	4.29%
Patient 8 (12.5 mm)	100	0.14%	97.33	2.95%
Patient 9 (15 mm)	99.3	-0.99%	100	1.73%
Patient 10 (20 mm)	100	0.37%	99.98	-2.11%
Patient 11 (20 mm)	100	0.40%	98.41	-0.49%
Patient 12 (25 mm)	100	0.86%	99.54	-3.32%
Mean ± **SD (Correlation with absolute % difference)	99.97 ± 0.54	4.01 ± 4.21	97.44 ± 3.73	2.31 ± 1.25
Spearman correlation (*ρ*)	0.06	-0.87	0.04	0.04
*p-value* (Correlation with absolute % difference)	0.847	*p* < 0.0001	0.892	0.913

*Phantom-based QA quantified the gamma passing rate using GAFchromic EBT3 film and measured %dose difference using Exradin A16 ionization chamber.

**SD, Standard deviation.

**Figure 4:**
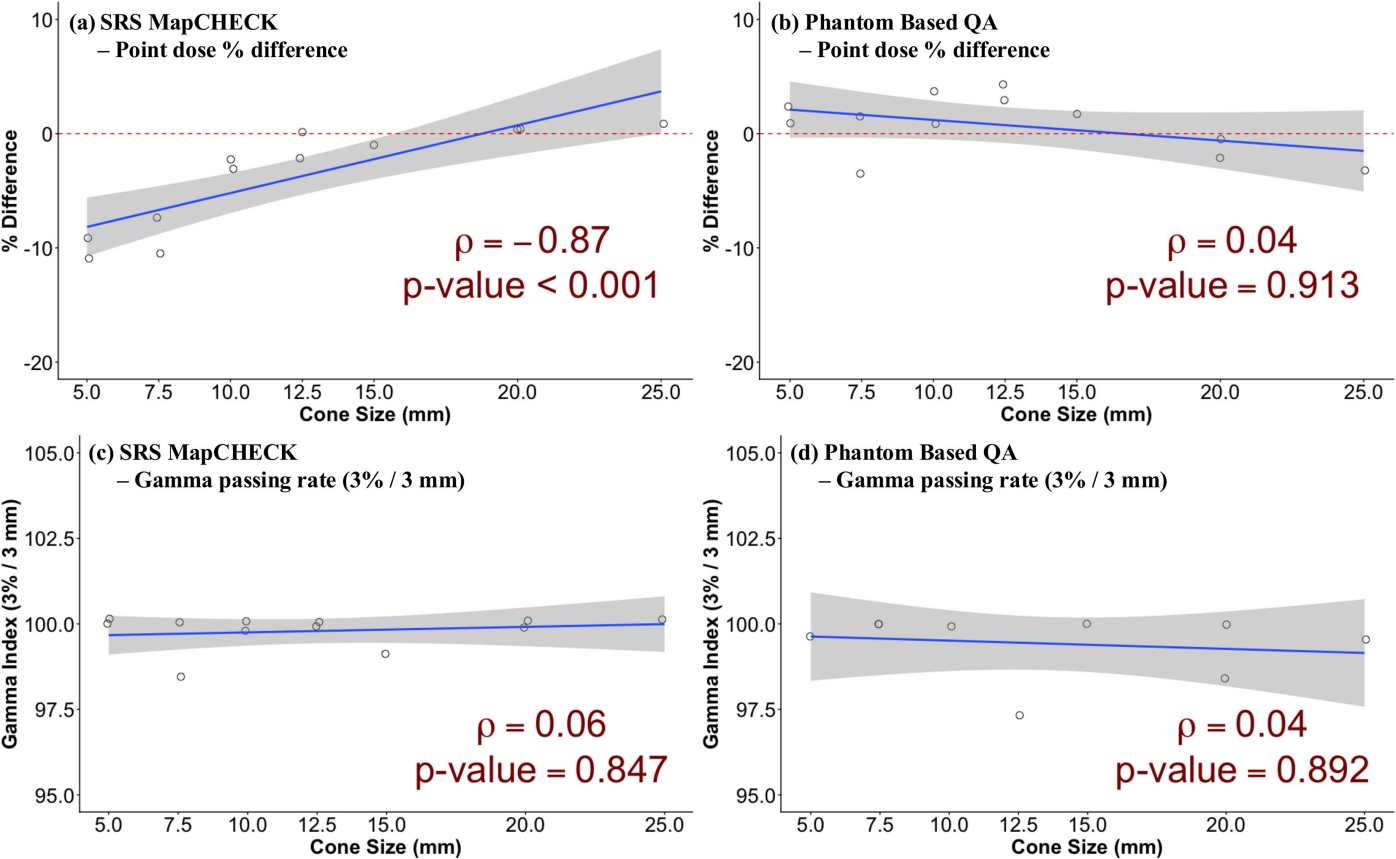
Scatter plots showing the relationship between fixed cone size (mm) and the point dose percentage difference (%) measured by **(a)** SRS MapCHECK and **(b)** phantom based QA using Exradin A16 ionization chamber relative to TPS calculation for 12 patient plans, and between fixed cone size (mm) and gamma passing rate (3%/3 mm) for **(c)** SRS MapCHECK and **(d)** phantom based QA using GAFchromic EBT3 film for 12 patient plans.

Based on the numerical results, the statistical analyses were performed to elaborate on the correlation between the fixed cone size and dosimetric accuracy of the SRS MapCHECK. As shown in **Table 1**; **Figure 4**, the analysis revealed a strong, statistically significant negative correlation (*ρ* = -0.87, *p-value* < 0.0001) between the cone size and the dosimetric error. This confirms that the dosimetric discrepancy significantly increases as the cone size decreases. For the remaining indices, including the gamma passing rate from the SRS MapCHECK measurements and both point dose error and gamma passing rate from the phantom-based PSQA, no statistically significant correlations were found. Additionally, a group analysis was performed to find an effective threshold for the fixed cone size in assessing the clinical applicability of the SRS MapCHECK in the CyberKnife system. As shown in the **Table 2**, the patient cohort was divided into groups to compare the mean percentage difference using an unpaired t-test. When comparing Group 1 (5, 7.5, and 10 mm; mean = -7.21 ± 3.74) and Group 2 (12.5, 15, 20, and 25 mm; mean = -0.23 ± 1.13), a highly significant difference was found (*p-value* = 0.0049). A similar significant difference was observed when comparing Group 3 (5, 7.5, 10, and 12.5 mm; mean = -5.66 ± 4.31) and Group 4 (15, 20, and 25 mm; mean = -0.16 ± 0.80), with a *p-value* of 0.0062. This robustly demonstrates that the dosimetric error is significantly greater for small cones, regardless of the precise grouping cutoff. Based on the results of the analyses, however, the fixed cone size of 12.5 mm appears to be the threshold for the safe use of the SRS MapCHECK in the CyberKnife system.

**Table 2:** Comparison of mean percent difference (± standard deviation) using unpaired t-tests between patient groups stratified by cone size.

Case size	# of patient	Mean ± standard deviation	*P-value*
Group 1 (5, 7.5, and 10 mm)	6	-7.21 ± 3.74	0.0049
Group 2 (12.5, 15, 20, and 25 mm)	6	-0.23 ± 1.13	
Group 3 (5, 7.5, 10, and 12.5 mm)	8	-5.66 ± 4.31	0.0062
Group 4 (15, 20, and 25 mm)	4	-0.16 ± 0.80	

Two different stratification methods were used: Group 1 (≤10 mm) vs Group 2 (>10 mm) and Group 3 (≤12.5 mm) vs Group 4 (>12.5 mm).

### 3.2 Measurement of static field delivery

**Figure 5:**
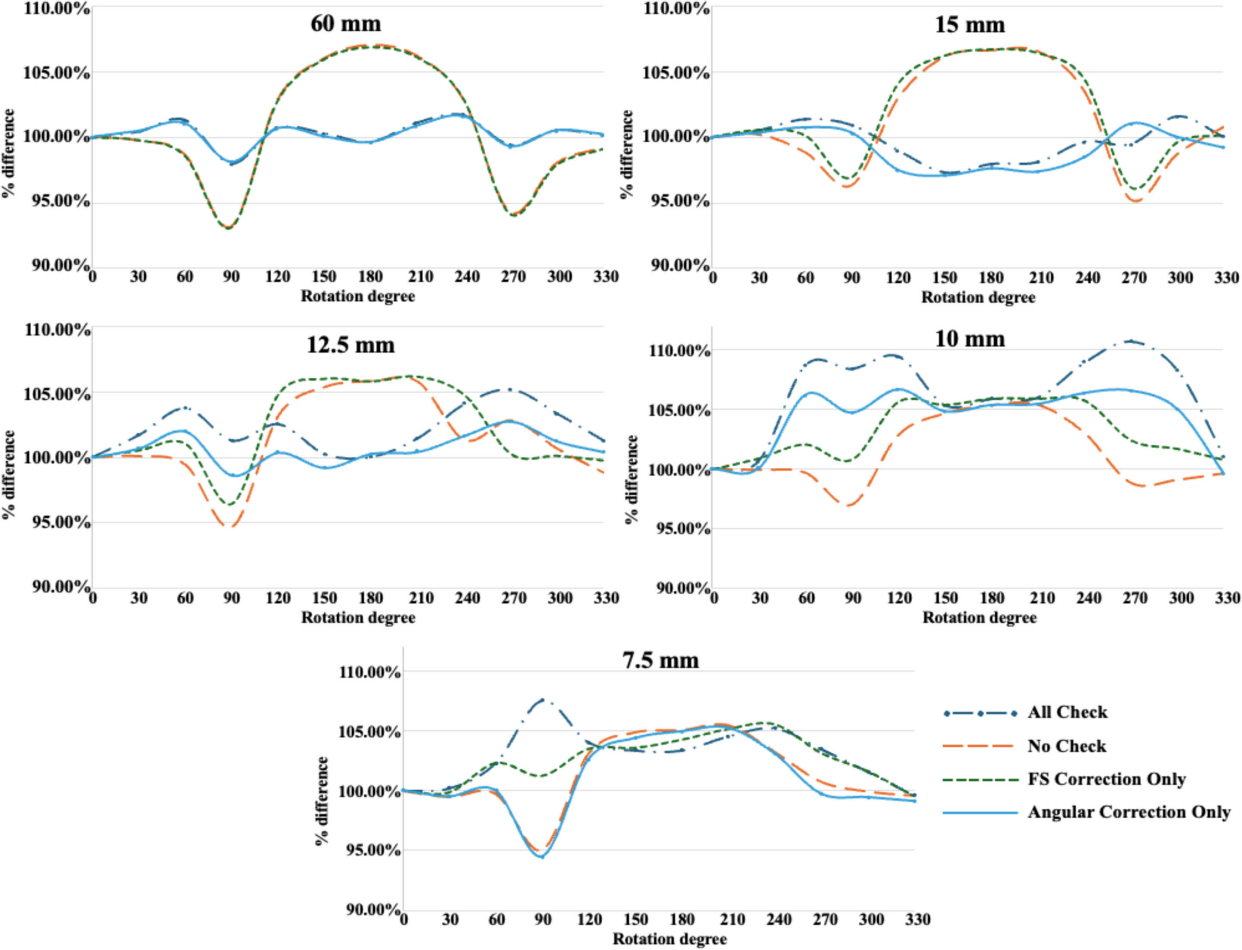
Measured dose across different robotic arm angles with different cone sizes (60, 15, 12.5, 10, and 7.5 mm) when different correction factors were applied (All check, No check, Field size correction only, and Angular correction only).

**Figure 5** illustrates the SRS MapCHECK measured dose across different robotic arm rotating angles (0° to 330° at 30° intervals) for different fixed cone sizes (7.5, 10, 12.5, 15 and 60 mm). Detailed numerical results are provided in **Supplementary Table S1** of the Supplementary Information. The measurements were normalized to the dose measured at 0°for each set-up. Importantly, unlike the PSQA analysis, the field size and angular correction factors were manipulated to be turned on and off to analyze their effect on dosimetry accuracy. Commonly, under the “No check” condition, in which all the correction factors were off, dose fluctuations exceeded 5% across different robotic arm angles for all fixed cone sizes. Turning on all corrections (“All check”) significantly improved stability in dose measurement across different angles for the 15 and 60 mm fixed cones, whereas the fluctuations remained unchanged for the small fixed cones smaller than or equal to 12.5 mm. Enabling all corrections even worsened the dose stability for the 10 and 7.5 mm cones. For the 12.5 mm fixed cone, dose consistency was enhanced when only the angular correction factor was active. The field size correction did not function when the fixed cone sizes were relatively large (15 and 60 mm), while it began working as the field size decreased, although it introduced inaccuracies. The angular correction factor had a bigger impact on stability in dose measurements than the field size correction factor. However, dose measurements with the angular correction factor on behaved the same as the “No check” for the 7.5 mm fixed cone, indicating its ineffectiveness for very small fields.

## 4 Discussion

The CyberKnife system relies on its unique non-coplanar and non-isocentric radiotherapy approach, which allows for precise dose delivery to small target volumes in SRS/SBRT. In particular, the non-isocentric treatment scheme necessitates rigorous dose verification, while routine periodic QA tasks do not include this verification. In this circumstance, the SRS MapCHECK with compact size and high-resolution 2D array detectors was expected to simplify and enhance the PSQA process for CyberKnife. As the target volume was small in most cases, the gamma passing rates at the 3%/3 mm criterion were acceptable for all fixed cone sizes. However, point dose measurements revealed large discrepancies relative to TPS calculations, particularly for small fields. Notably, the dose differences became more pronounced as the cone size decreased, showing a strong and statistically significant negative correlation (spearman correlation *ρ* = -0.87, *p-value* < 0.0001). The errors were substantial for the small cones, reaching up to -10.92% for the 5 mm cone. Group analysis further confirmed this trend; cones ≤12.5 mm exhibited a significantly larger mean under-measurement (-5.66 ± 4.31) compared to cone size ≥ 15 mm (-0.16 ± 0.80, *p-value* = 0.0062). Importantly, throughout the comparative analysis of our in-house PSQA procedure, the dosimetry results remained clinically acceptable in both point dose measurements and gamma passing rates. This implies that the discrepancies observed with SRS MapCHECK were not due to inaccuracies in the TPS calculations. To investigate these discrepancies, this study evaluated the effectiveness of the correction factors through static dose delivery experiments, while varying gantry angles and fixed cone sizes. The analysis revealed that the correction function of SRS MapCHECK was particularly ineffective for small fields. While the field size correction factor began to work as the field size decreased, the accuracy became worse for very small fixed cones. The angular correction factor performed slightly better across the field sizes, yet its overall impact remained limited, as the corrected values tended to converge with those obtained without any correction factors. Enabling both the field size and angular correction factors improved dose stability for relatively large cone sizes (e.g., >12.5 mm), but had minimal or even adverse effects on smaller cones (<12.5 mm), consistent with the PSQA results. Specifically, neither correction factor worked reliably for fixed cones smaller than 12.5 mm.

Our in-house PSQA procedure, as stated above, was based on phantom-based measurements using an Exradin A16 ionization chamber and film dosimetry. In most treatment cases, the small target volumes made it difficult to perform point dose and film dosimetry simultaneously. Compared to this conventional, time-consuming phantom-based PSQA, the SRS MapCHECK-based PSQA was expected to dramatically improve efficiency. However, due to dosimetry inaccuracies observed at small fixed cones (<12.5 mm), it failed to achieve this goal. Therefore, the SRS MapCHECK system would be clinically applicable to treatment plans using fixed cones ≥12.5 mm, while phantom-based PSQA should remain preferable for smaller fields to ensure patient safety.

This study has several limitations to be discussed. First, this study was conducted using coplanar gantry rotating angles for static dose verification, which did not fully replicate the complex non-coplanar beam trajectories unique to CyberKnife treatments. In practice, it was nearly impossible to position and set-up robotic arms at certain tilted angles combined with specific gantry angles, which would require an excessive number of configurations and a significant amount of time to cover all possible combinations of non-coplanar delivery geometries. Stansook et al. conducted a similar study involving various gantry angles and field sizes to test the 2D array detector, excluding the couch angle rotation (non-coplanar scheme) (30). More recently, Kawata et al., reported that the detector response highly depended on gantry rotation angle and field size, while the deviations from non-coplanar settings (couch rotation) remained within 2.5% (31). Thus, it is reasonable to assume that the point dose errors would not differ substantially under fully non-coplanar PSQA conditions. More importantly, the purpose of the static field test in this work was intended to investigate and identify the sources of dosimetric errors in the SRS MapCHECK. We found that significant discrepancies arise even in this simplified geometry (gantry angle rotation), originating from both angular and field size correction factors at small fixed cones. In fact, the PSQA results from 12 patient cases with different fixed cones were consistent with those of the static dose delivery test, showing a similar degree of point dose error across fixed cone sizes. Second, the evaluation was conducted on a limited cohort of 12 patient cases. While larger patient data would provide more definitive conclusions, the results from 12 patient cases demonstrated statistically robust validation of the SRS MapCHECK point dose errors across different fixed cone sizes, enabling rigorous correlation and group analyses. Notably, this cohort ultimately helped clarify the appropriate fixed cone sizes for the safe use of the SRS MapCHECK with the CyberKnife system. In addition, this study was limited to fixed cone sizes between 5 and 25 mm and did not evaluate Iris collimators or MLC-based treatments, which may limit the generalizability of the findings. Furthermore, the SRS MapCHECK system provides only two-dimensional dose verification, which may not fully reflect the three-dimensional nature of dose distributions in SRS/SBRT.

## 5 Conclusion

This study investigated the effectiveness of SRS MapCHECK for PSQA of the CyberKnife system. The findings revealed that PSQA results from SRS MapCHECK exhibited increasing deviations from TPS calculations as the fixed cone size decreased. Analysis of the static dose delivery test attributed these discrepancies to unwanted operations of the correction factors for beam angle and field size. Overall, this work demonstrated that SRS MapCHECK offers accurate and reliable PSQA of the CyberKnife system when the fixed cone size is greater than or equal to 12.5 mm.

## Data Availability

The original contributions presented in the study are included in the article/**Supplementary Material**. Further inquiries can be directed to the corresponding authors.
