# Supplementary material for: Validation of SRS MapCHECK for CyberKnife patient-specific quality assurance: challenges with small cone sizes
Source: Front Oncol. 2026 Jan 8;15:1667108. doi: 10.3389/fonc.2025.1667108 (PMC12823868; doi:10.3389/fonc.2025.1667108)
Supplement: Supplementary file 1 [file Table1.docx]

Supplementary Material

# Supplementary Table

**Table S1.** Dose and percentage difference across various cone sizes and rotational angles with different correction methods (All check, No check, Field size correction only, and angular correction only)

| **Cone size** | **Rotation degree** | **All check** | | **No check** | | **FS correction only** | | **Angular correction only** | |
| --- | --- | --- | --- | --- | --- | --- | --- | --- | --- |
|  |  | **Dose (cGy)** | **% Diff.** | **Dose (cGy)** | **% Diff.** | **Dose (cGy)** | **% Diff.** | **Dose (cGy)** | **% Diff.** |
| **60 mm** | 0 | 85.739 | 100.00% | 85.547 | 100.00% | 85.762 | 100.00% | 85.598 | 100.00% |
|  | 30 | 86.097 | 100.42% | 85.318 | 99.73% | 85.586 | 99.79% | 85.985 | 100.45% |
|  | 60 | 86.887 | 101.34% | 84.428 | 98.69% | 84.581 | 98.62% | 86.489 | 101.04% |
|  | 90 | 84.005 | 97.98% | 79.718 | 93.19% | 79.865 | 93.12% | 83.963 | 98.09% |
|  | 120 | 86.347 | 100.71% | 87.967 | 102.83% | 88.127 | 102.76% | 86.166 | 100.66% |
|  | 150 | 86.017 | 100.32% | 90.650 | 105.97% | 90.852 | 105.93% | 85.634 | 100.04% |
|  | 180 | 85.443 | 99.65% | 91.531 | 106.99% | 91.647 | 106.86% | 85.253 | 99.60% |
|  | 210 | 86.695 | 101.12% | 90.817 | 106.16% | 90.989 | 106.09% | 86.326 | 100.85% |
|  | 240 | 87.195 | 101.70% | 87.973 | 102.84% | 88.276 | 102.93% | 86.954 | 101.58% |
|  | 270 | 85.242 | 99.42% | 80.667 | 94.30% | 80.766 | 94.17% | 84.987 | 99.29% |
|  | 300 | 86.204 | 100.54% | 83.846 | 98.01% | 83.961 | 97.90% | 86.035 | 100.51% |
|  | 330 | 85.878 | 100.16% | 84.873 | 99.21% | 85.001 | 99.11% | 85.775 | 100.21% |
| **15 mm** | 0 | 72.861 | 100.00% | 72.425 | 100.00% | 71.744 | 100.00% | 73.430 | 100.00% |
|  | 30 | 73.258 | 100.54% | 72.577 | 100.21% | 72.110 | 100.51% | 73.681 | 100.34% |
|  | 60 | 73.881 | 101.40% | 71.584 | 98.84% | 71.809 | 100.09% | 73.972 | 100.74% |
|  | 90 | 73.549 | 100.94% | 69.775 | 96.34% | 69.536 | 96.92% | 73.671 | 100.33% |
|  | 120 | 72.121 | 98.99% | 74.615 | 103.02% | 74.672 | 104.08% | 71.608 | 97.52% |
|  | 150 | 70.897 | 97.30% | 76.890 | 106.16% | 76.186 | 106.19% | 71.315 | 97.12% |
|  | 180 | 71.375 | 97.96% | 77.233 | 106.64% | 76.523 | 106.66% | 71.707 | 97.65% |
|  | 210 | 71.477 | 98.10% | 77.183 | 106.57% | 76.321 | 106.38% | 71.528 | 97.41% |
|  | 240 | 72.622 | 99.67% | 74.972 | 103.52% | 74.894 | 104.39% | 72.320 | 98.49% |
|  | 270 | 72.489 | 99.49% | 68.972 | 95.23% | 68.972 | 96.14% | 74.187 | 101.03% |
|  | 300 | 74.044 | 101.62% | 71.501 | 98.72% | 71.445 | 99.58% | 73.439 | 100.01% |
|  | 330 | 72.875 | 100.02% | 72.996 | 100.79% | 71.846 | 100.14% | 72.848 | 99.21% |
| **12.5 mm** | 0 | 68.834 | 100.00% | 70.471 | 100.00% | 68.866 | 100.00% | 70.617 | 100.00% |
|  | 30 | 69.994 | 101.69% | 70.541 | 100.10% | 69.210 | 100.50% | 71.111 | 100.70% |
|  | 60 | 71.412 | 103.75% | 70.062 | 99.42% | 69.552 | 101.00% | 72.013 | 101.98% |
|  | 90 | 69.704 | 101.26% | 66.719 | 94.68% | 66.413 | 96.44% | 69.657 | 98.64% |
|  | 120 | 70.564 | 102.51% | 72.746 | 103.23% | 72.181 | 104.81% | 70.887 | 100.38% |
|  | 150 | 68.979 | 100.21% | 74.279 | 105.40% | 72.975 | 105.97% | 70.068 | 99.22% |
|  | 180 | 68.832 | 100.00% | 74.577 | 105.83% | 72.838 | 105.77% | 70.821 | 100.29% |
|  | 210 | 69.836 | 101.46% | 74.541 | 105.78% | 73.078 | 106.12% | 70.927 | 100.44% |
|  | 240 | 71.626 | 104.06% | 71.411 | 101.33% | 72.107 | 104.71% | 71.789 | 101.66% |
|  | 270 | 72.400 | 105.18% | 72.467 | 102.83% | 69.016 | 100.22% | 72.556 | 102.74% |
|  | 300 | 71.112 | 103.31% | 70.926 | 100.65% | 68.927 | 100.09% | 71.479 | 101.22% |
|  | 330 | 69.685 | 101.24% | 69.636 | 98.82% | 68.683 | 99.73% | 70.917 | 100.42% |
| **10 mm** | 0 | 62.198 | 100.00% | 67.170 | 100.00% | 64.384 | 100.00% | 64.812 | 100.00% |
|  | 30 | 62.633 | 100.70% | 67.124 | 99.93% | 64.979 | 100.92% | 64.868 | 100.09% |
|  | 60 | 67.635 | 108.74% | 66.967 | 99.70% | 65.729 | 102.09% | 68.849 | 106.23% |
|  | 90 | 67.447 | 108.44% | 65.169 | 97.02% | 64.914 | 100.82% | 67.878 | 104.73% |
|  | 120 | 68.102 | 109.49% | 69.100 | 102.87% | 68.040 | 105.68% | 69.143 | 106.68% |
|  | 150 | 65.539 | 105.37% | 70.306 | 104.67% | 67.873 | 105.42% | 67.949 | 104.84% |
|  | 180 | 65.867 | 105.90% | 70.785 | 105.38% | 68.208 | 105.94% | 68.294 | 105.37% |
|  | 210 | 65.942 | 106.02% | 70.805 | 105.41% | 68.210 | 105.94% | 68.345 | 105.45% |
|  | 240 | 67.766 | 108.95% | 69.221 | 103.05% | 68.120 | 105.80% | 68.950 | 106.38% |
|  | 270 | 68.879 | 110.74% | 66.388 | 98.84% | 65.961 | 102.45% | 69.077 | 106.58% |
|  | 300 | 67.379 | 108.33% | 66.565 | 99.10% | 65.500 | 101.73% | 68.071 | 105.03% |
|  | 330 | 62.875 | 101.09% | 66.934 | 99.65% | 64.899 | 100.80% | 64.580 | 99.64% |
| **7.5 mm** | 0 | 92.558 | 100.00% | 100.348 | 100.00% | 95.975 | 100.00% | 97.063 | 100.00% |
|  | 30 | 92.740 | 100.20% | 99.840 | 99.49% | 95.814 | 99.83% | 96.575 | 99.50% |
|  | 60 | 94.692 | 102.30% | 100.108 | 99.76% | 98.165 | 102.28% | 97.049 | 99.99% |
|  | 90 | 99.569 | 107.57% | 95.416 | 95.08% | 97.133 | 101.21% | 91.696 | 94.47% |
|  | 120 | 96.313 | 104.06% | 103.482 | 103.12% | 99.299 | 103.46% | 99.606 | 102.62% |
|  | 150 | 95.684 | 103.38% | 105.241 | 104.88% | 99.376 | 103.54% | 101.328 | 104.39% |
|  | 180 | 95.680 | 103.37% | 105.432 | 105.07% | 100.015 | 104.21% | 101.869 | 104.95% |
|  | 210 | 96.783 | 104.56% | 105.859 | 105.49% | 100.873 | 105.10% | 102.157 | 105.25% |
|  | 240 | 97.407 | 105.24% | 103.615 | 103.26% | 101.252 | 105.50% | 100.067 | 103.10% |
|  | 270 | 95.801 | 103.50% | 101.119 | 100.77% | 98.962 | 103.11% | 96.792 | 99.72% |
|  | 300 | 94.052 | 101.61% | 100.294 | 99.95% | 97.511 | 101.60% | 96.523 | 99.44% |
|  | 330 | 92.178 | 99.59% | 99.949 | 99.60% | 95.510 | 99.52% | 96.200 | 99.11% |
